# Subjective Memory Complaint and Depressive Symptoms among Older Adults in Portugal

**DOI:** 10.1155/2015/296581

**Published:** 2015-12-31

**Authors:** Mónica Sousa, Anabela Pereira, Rui Costa

**Affiliations:** Aveiro University, Campus Universitário de Santiago, 3810-193 Aveiro, Portugal

## Abstract

*Background*. Older adults report subjective memory complaints (SMCs) but whether these are related to depression remains controversial. In this study we investigated the relationship between the SMCs and depression and their predictors in a sample of old adults.* Methods*. This cross-sectional study enrolled 620 participants aged 55 to 96 years (74.04 ± 10.41). Outcome measures included a sociodemographic and clinical questionnaire, a SMC scale (QSM), a Geriatric Depression Scale (GDS), a Mini-Mental Status Examination (MMSE), and a Montreal Cognitive Assessment (MoCA).* Results*. The QSM mean total score for the main results suggests that SMCs are higher in old adults with depressed symptoms, comparatively to nondepressed old adults. The GDS scores were positively associated with QSM but negatively associated with education, MMSE, and MoCA. GDS scores predicted almost 63.4% of variance. Scores on QSM and MoCA are significantly predicted by depression symptomatology.* Conclusion*. Depression symptoms, lower education level, and older age may be crucial to the comprehension of SMCs. The present study suggested that depression might play a role in the SMCs of the older adults and its treatment should be considered.

## 1. Introduction

The aging process is complex and dynamic. For this reasons the cognitive performance over the lifespan is a heterogeneous process, associated with interindividual variability (diversity) and intraindividual variability (dispersion) [1, 2]. This complexity is also present in the controversial topic of the subjective memory complaint (SMC).

The SMCs are complains about memory problems of people in the absence, or not, of cognitive impairment [3]. Previous Portuguese studies have reported that 75.9% [4] and/or 80.4% [5] of older adults complain of memory problems.

Based on several meta-analyses, systematic reviews, and research studies, evidence that suggests that SMCs are associated with an increased risk of dementia is inconclusive [3]. Most postulate that SMCs increase with advancing of age, are a core cognitive criteria for the early diagnosis of MCI and prodromal Alzheimer disease (AD), and have value as a predictor of dementia [6, 7]. On the other hand, it is considered that SMC could not predict future conversion to dementia [8]. A Portuguese study shows that in a memory clinic setting the SMCs have no differences in the conversion to dementia [9]. Notably, a recent systematic review shows that approximately 2.3%–6.6% of older adults with SMCs will develop mild cognitive impairment (MCI) and dementia per year [10]. Therefore, it is believed that there is no treatment that can stop the progress of dementia, but with the early detection of signs the medical treatment can slow down this disease process [11].

The presence of preclinical AD in individuals with SMCs reinforces the importance of identifying modifiable risk factors associated with cognitive decline in middle-aged populations [12]. The recent study of the World Health Organization (WHO) [13] reveled that depression in the community is around 5%. In late life, depression is common [14]; however it is not a natural part of aging. There is still a dispute over whether SMCs reflect depressive disorder [14–17], rather than early memory impairment [7], or if depression can be an early marker of brain changes that characterize dementia [12].

Besides age, sex, and level of education, the most prominent factor strongly associated with SMC is depression [14–17]. Although SMC is not associated with greater risk of mortality, it was strongly associated with depression [14, 18]. According to Singh-Manoux [18], reporting to the doctor about memory complain was related to risk of mortality. However, this active seek for help can reflect more worries about memory [8].

There is a consistent evidence that untreated depression may lead to physical, cognitive, functional, and social impairment, as well as decreased quality of life. Appropriate treatment may allow the curing of depression; however the effect of this treatment on subsequent cognitive functioning is not well understood [12].

The present study explores how old adults with SMC and depressive or nondepressive symptoms rate their levels of memory complaint. In Portuguese older adults, there was a particular interest in whether SMCs are associated with poor performance in screening tests such as the Mini-Mental Status Examination (MMSE) [19] and the Montreal Cognitive Assessment (MoCA) [20] and whether SMCs are associated with measures of gender, age, education, and depression was also investigated in order to examine the factors that influence these SMCs. Moreover, to the best of our knowledge, most of the Portuguese research investigating the relationship between SMC and depression symptoms generally use homogenous or clinical patients samples [4] and excluded patients with major depression [5, 9]. For that reason, the central question of this study was to verify the difference in older adults with depressive symptomatology through the comparison with older adults without depressive symptomatology. It was further hypothesized that SMC is related to depression and we expected that older adults with depression are of older age and female, have a lower education level, and show lower scores in screenings tests.

## 2. Methods

### 2.1. Study Design and Participants

This is a cross-sectional study with a convenience sample recruited at the local health center and nursing homes of different regions of Portugal (Coimbra and island of Madeira) where it was conducted.

The inclusion criteria included were old adults with age of 55 years and older willing to participate in the present study. The exclusion criteria were (i) age less than or equal to 54 years, (ii) presence of neurological or psychiatric disorder, (iii) chronic alcohol or drug abuse, (iv) inability to understand and cooperate, and (v) being nonnative Portuguese.

Informed consent was obtained from all participants and the study received ethical approval from the University of Aveiro and Institutional Ethics Committee.

### 2.2. Procedures

A semi-structured interview was conducted by a trained psychologist to record sociodemographic and clinical information, psychiatric and neurological history, past habits, and medical history. A standard protocol comprising test and scales of neuropsychological assessment was carried out.

#### 2.2.1. Memory Complaint

The Portuguese version of SMC scale (QSM) [21] was used for the assessment of SMC. Scores ≥ 4 indicate clinically significant SMC.

#### 2.2.2. Depressive Symptoms

The presence of depressive mood was evaluated using Geriatric Depression Scale (GDS) [22]. A score < 10 in the GDS was used to consider the absence of depression symptoms.

#### 2.2.3. Cognitive Domain

The global cognitive status was assessed with the MMSE [19] and the MoCA [20], following the respective correspondence of the validation studies for Portuguese population participants scoring.

## 3. Statistical Analysis

Descriptive statistics are presented as means with standard deviations for continuous variables and as percentages for categorical variables. The analysis of differences between the two groups (nondepressed and depressed) was conducted by Chi-squared and independent *t*-tests. We examined the Pearson's correlation coefficients for the associations between demographic variables (age, gender, and education), MMSE, and MoCA, with the GDS and QSM total score. Linear regression models were used to predict SMC and depression performance scores adjusted by independent variables, namely, age, gender, education, MMSE, and MoCA, considering the Enter method.

All tests were two-tailed and a *p* value < 0.05 was assumed as statistically significant.

We performed the statistical analysis with the Statistical Package for the Social Sciences (SPSS) v22.0 package for Windows.

## 4. Results

Table 1 provides the sample characteristics and results of the neuropsychological assessment. The 620 participants included 449 women and 171 men with a mean age of 74.04 years (SD ± 10.41). The mean education level of the entire group was 3.61 ± 3.38 years. A total of 548 (88.4%) of participants only completed primary school or less, and 72 (11.6%) had secondary school education or higher. The mean total score of the SMC was 7.69 ± 4.28 and most of the participants had SMC (78.9%). Clinically significant depression symptoms were present in 46.3% (*n* = 287) of the participants.

There is no statistically significant association between gender and depression symptoms (*χ* ^2^(1) = 2.723, *p* = 0.09). Depression symptoms were more frequent in SMC participants (*χ* ^2^(1) = 46.712, *p* = 0.00) with lower education level (*χ* ^2^(5) = 44.370, *p* = 0.00; *t*(618) = 3.833, *p* = 0.00) and older age (*t*(610.82) = −3.965, *p* = 0.00). The depressed participants showed significant improvement in QSM score (*t*(618) = 17.981, *p* = 0.00) but a significant decrease in MMSE score (*t*(618) = −13.408, *p* = 0.00) and MoCA *t*(618) = 30.722, *p* = 0.00 (Table 1).

Table 2 shows that there were no significant differences between both groups only in items (5) (*Do you often use notes to avoid forgetting things?*; *χ* ^2^(2) = 44.370, *p* = 0.18) and (7) (*Did you ever lose your way in neighborhood?*; *χ* ^2^(1) = 0.009, *p* = 0.92). The analysis of the other items suggests that depressed patients had generally answered the last option of the scoring.

**Figure 1:**
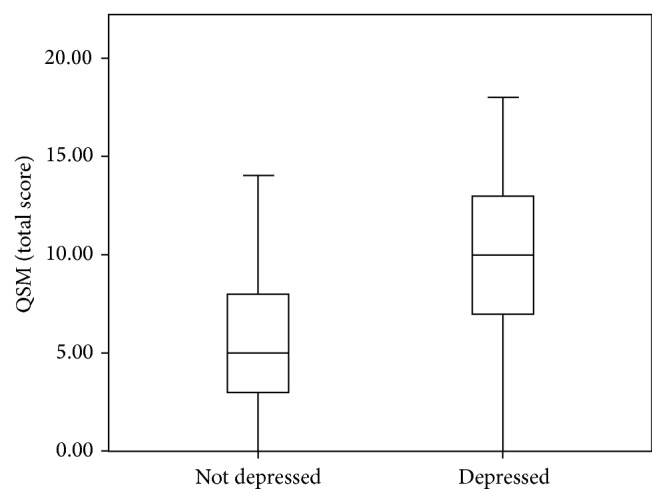
Total QSM score in the depressed and not depressed old adults.

As indicated in Figure 1, old adults with depression had higher scores on total SMC (9.87 ± 4.29; 0–14), comparatively to old adults without depression (5.80 ± 3.26; 0–18). Only one (0.3%) participant without depression symptoms and eight (2.8%) depression participants reported no memory complaint, in other words, had QSM equal to 0.

GDS score obtained significant weak negative correlation with education (*r* = −0.29, *p* < 0.001), MMSE (*r* = −0.43, *p* < 0.001), and MoCa (*r* = −0.49, *p* < 0.001) and only a significant weak positive correlation with age (*r* = 0.24, *p* < 0.001). QSM score showed a significant, weak, and positive correlation with age (*r* = 0.14, *p* < 0.001) and moderate positive correlation with GDS (*r* = 0.54, *p* < 0.001). Education (*r* = −0.13, *p* < 0.001), MMSE, and MoCa (*r* = −0.34, *p* < 0.001) have significant weak negative correlation with QSM (Table 3).

Two multivariate logistic regressions were performed to identify the predictors of QSM and GDS scores. According to the results shown in Table 4, the QSM score was only influenced by education (*β* = 0.14, 95% confidence interval (CI) = −0.823–0.475), MMSE (*β* = −0.11, 95% CI = 0.034–0.241), and GDS scale scores (*β* = 0.40, 95% CI = −0.112–0.59). Age, gender, and MoCA were not influenced by QSM score (Table 3). The GDS performance were predicted by MoCA (*β* = −0.402, 95% CI = −0.341–−0.162) and QSM (*β* = −0.419, 95% CI = −0.408–0.561). These logistic regression models predicted 63.4% of total variations of GDS score and 31% of SMC score (Table 3).

## 5. Discussion

In the present study, we analyzed SMC and depression, and their relationship to sociodemographic and to the scores in MMSE and MoCA. The initial hypothesis that SMCs would be more reported by depressed old adults, as compared to nondepressed, was confirmed. However, in both groups, few participants had zero in the QSM total score. These findings are consistent with previous research on community samples, in which few participants also reported none memory difficulties measured by QSM [8].

On whole sample, the frequencies of SMC and depression are in line with those observed in other studies [14, 15, 18], highlighting the higher frequencies in the Portuguese old adults, independently of the characteristics of participants and settings where they are recruited [4, 5, 8, 9, 23].

Several studies have shown that older adults with depressive symptoms had significantly more SMCs, compared to older adults without these symptoms [15, 18, 24]. In this study, thus, depressive symptoms appear to be an important predictor of SMC and depression, age, education, and both screening instruments were significantly associated with QSM score. Despite the weak association, this result is analogous to the Portuguese studies [4, 23], observing positive correlations of depression with QSM score, and emphasizes that the lower cognitive performance influences the reports of memory dysfunction [14, 16].

The main findings were that participants without depression had more education, had higher scores on the MMSE and the MoCA, and had slightly minor QSM score than the participants with depression [14]. The poor cognitive function and inclination to SMC might be a reflection of a negative mental status and cognitive changes produced by anxiety and depression [14]. Therefore, overall, the Portuguese population of women over 64 years who completed primary education have a higher longevity [25, 26]. This research, based on self-reported measures of SMC and in a convenience sampling, illustrated this phenomenon. For this reason, these factors should be considered in the interpretation of the results.

Concerning the gender, no statistically significant differences were found for both SMC and depression symptoms. The influence of gender is not clear. Studies have demonstrated that there are no differences [8, 23], others that males have higher number of complaints [24], and others that women are at great risk for SMC [16].

Contrarily to previous research, in QSM items (5) (*Do you often use notes to avoid forgetting things*?) and (7) (*Did you ever lose your way in neighborhood*?), independently of whether depressed or nondepressed, few participants answered positively. Assessing the SMC with the same instrument [21], participants who were nonconverters to dementia had higher scores in item (5) [9] and participants from clinical and community sample also tend to score lower in item (7) [8]. A recent study performed in our population revealed the same higher option zero (0) in items (5) and (7) [5]. It also demonstrated that item (5) increased with the level of education and may be related to cognitive reserve and external strategies (listing dates or using schedules) [5].

In addition, the controversy between SMC and depression emphasizes that SMCs may have clinical usability to identify early cognitive changes self-described by old people [3, 6] but not so far detected in neuropsychological assessment. It also emphasizes that that depression can also increase the risk for dementia [12]. This study has significant implications for clinical practice, namely, the SMCs which should be considered clinically meaningful because they may have the potential to identify depressive symptoms.

Other possible limitations of the present investigation were the convenience sampling and the use of two cognitive screening tests. Future studies should include a larger sample that represents the Portuguese population, adopt random sampling, and should be evaluated with a comprehensive neuropsychological battery. However, the MMSE and MoCA were widely brief instruments that can provide important cognitive screening and are cost-effective for the clinical evaluation of adults' cognitive state [27]. This cross-sectional study might not provide causal information among variables and the majority of the sample was four years of education, opening the possibility to the presence of false positives. For this reason, we suggest that future studies should have a longitudinal design to deeply identify this causal relationship.

## 6. Conclusion

Our findings suggest that Portuguese old adults with age of 55 and older experience clinically significant depression symptoms and, as their age advances, lower education and lower cognitive function were significant predictors of the SMC. Approximately 78.9% of participants report significant SMC, with an increase form not depressed patients to depressed patients.

Based on these findings, we recommend that the clinicians, frequented by old adults with complaints of memory problems who seek help, should consider the different preventative measures and interventions that can be adopted to delay or reverse depression, and consequently the SMCs, because this kind of complaints can be part of a scenario in which mood disorder is a symptom.

## Figures and Tables

**Table 1:** Demographics and test scores of the study groups.

	Whole sample (*n* = 620)	GDS		*p*
		Not depressed (GDS < 10; *n* = 333)	Depressed (GDS ≥ 11; *n* = 287)	
Age (years) M *± *SD	74.04 ± 10.41	72.52 ± 10.48	75.80 ± 10.06	0.00^b^
Female *n* (%)	449 (72.4)	232 (69.7)	217 (75.6)	0.09^a^
Education (years) M *± *SD	3.61 ± 3.38	4.27 ± 3.55	2.85 ± 2.99	0.00^b^
No education completed *n* (%)	178 (28.7)	61 (18.3)	117 (40.8)	0.00^a^
Primary school *n* (%)	370 (59.7)	249 (74.8)	159 (55.4)	
Secondary school *n* (%)	56 (9)	9 (2.7)	9 (3.1)	
High school/university *n* (%)	16 (2.6)	14 (4.2)	2 (0.7)	
MMSE M *± *SD (range)	24.85 ± 5.61 (6–30)	26.61 ± 4.38	22.80 ± 6.17	0.00^b^
MoCA M *± *SD (range)	18.20 ± 7.93 (1–31)	20.97 ± 6.64	14.98 ± 8.08	0.00^b^
QSM M *± *SD (range)	7.69 ± 4.28 (0–18)	5.80 ± 3.26	9.87 ± 4.29	0.00^b^
Clinically significant SMC *n* (%)	489 (78.9)	228 (68.5)	261 (90.9)	0.00^a^
GDS M *± *SD (range)	9.28 ± 4.95 (0–20)			

MMSE: Mini-Mental State Examination; MoCA: Montreal Cognitive Assessment; QSM: Portuguese version of SMC scale; GDS: Geriatric Depression Scale.

^a^Chi-square test.

^b^Independent *t*-tests.

**Table 2:** Results of the QSM.

Item	Subscore range	Participants score answers, %		χ^2^	*p*
		Not depressed (GDS < 10; *n* = 333)	Depressed (GDS ≥ 11; *n* = 287)		
(1) Do you have any complaints concerning your memory?	0–3	0 = 6.9; 1 = 48.6; 2 = 26.4; 3 = 18	0 = 4.2; 1 = 19.5; 2 = 37.3; 3 = 39	69.541	0.00
(2) Do other people find you forgetful?	0–2	0 = 60.7; 1 = 27.3; 2 = 12	0 = 40.1; 1 = 31.7; 2 = 28.2	34.547	0.00
(3) Do you ever forget names of family members or friends?	0–3	0 = 69.4; 1 = 19.2; 2 = 9.6; 3 = 1.8	0 = 45.3; 1 = 19.5; 2 = 26.5; 3 = 8.7	55.253	0.00
(4) Do you often forget where things are left?	0–3	0 = 20.7; 1 = 51.7; 2 = 18.6; 3 = 9	0 = 25.1; 1 = 20.6; 2 = 28.9; 3 = 25.4	73.324	0.00
(5) Do you often use notes to avoid forgetting things?	0–2	0 = 84.4; 1 = 14.1; 2 = 1.5	0 = 82.9; 1 = 13.2; 2 = 3.8	3.371	0.18
(6) Do you ever have difficulties in finding particular words?	0-1	0 = 82; 1 = 18	0 = 59.9; 1 = 40.1	37.000	0.00
(7) Did you ever lose your way in neighborhood?	0-1	0 = 97; 1 = 3.0	0 = 96.9; 1 = 3.1	0.009	0.92
(8) Do you think more slowly than you used to?	0–2	0 = 28.2; 1 = 62.5; 2 = 9.3	0 = 11.8; 1 = 46.7; 2 = 41.5	92.862	0.00
(9) Do your thoughts ever become confused?	0–2	0 = 62.5; 1 = 29.7; 2 = 7.8	0 = 23.7; 1 = 31; 2 = 45.3	138.228	0.00
(10) Do you have concentration problems?	0–2	0 = 60.4; 1 = 30.6; 2 = 9	0 = 22; 1 = 36.6; 2 = 41.5	122.603	0.00

GDS: Geriatric Depression Scale; *χ* ^*2*^: Chi-square test.

Scoring of items (1), (3), and (4): 0: no; 1: yes, but no problem; 2: yes, problem; 3: yes, serious problem.

Scoring of items (2) and (5): 0: no; 1: yes, sometimes; 2: yes, often.

Scoring of items (6) and (7): 0: no; 1: yes.

Scoring of items (8)–(10): 0: no; 1: yes; 2: yes, serious problem.

**Table 3:** Correlation for the main variables and measures.

	GDS	QSM
Age	0.24^*∗∗*^	0.14^*∗∗*^
Education	−0.29^*∗∗*^	−0.13^*∗∗*^
MMSE	−0.43^*∗∗*^	−0.34^*∗∗*^
MoCA	−0.49^*∗∗*^	−0.34^*∗∗*^
GDS		0.54^*∗∗*^

MMSE: Mini-Mental State Examination; MoCA: Montreal Cognitive Assessment; QSM: Portuguese version of SMC scale; GDS: Geriatric Depression Scale.

^*∗∗*^ *p* < 0.001.

**Table 4:** Regression analysis of predictors of SMC and depression performance.

	QSM (*n* = 620)				GDS (*n* = 620)			
	*β*	(CI 95%)		*p*	*β*	(CI 95%)		*p*
Age	−0.008	3.790	10.632	0.649	−0.041	−0.056	0.017	0.296
Gender	−0.174	−0.042	0.026	0.599	−0.026	−0.980	0.413	0.425
Education	0.14	−0.823	0.475	0.009	−0.061	−0.200	0.023	0.118
MMSE	−0.11	0.034	0.241	0.035	0.074	−0.046	0.177	0.249
MoCA	−0.026	−0.215	−0.008	0.546	−0.402	−0.341	−0.162	0.000
GDS	0.40	−0.112	0.059	0.000				
QSM					0.419	0.408	0.561	0.000
*R* ^2^	31				63.4			
*F*(7.612) = 39.242. *p* < 0.001					*F*(6.613) = 68.659. *p* < 0.001			

*β*: beta coefficient; 95% CI: 95% confidence interval; MMSE: Mini-Mental State Examination; MoCA: Montreal Cognitive Assessment; QSM: Portuguese version of SMC scale; GDS: Geriatric Depression Scale; *R* ^2^: Nagelkerke *R* Square.

## References

[1] Vaughan L., Leng I., Dagenbach D. (2013). Intraindividual variability in domain-specific cognition and risk of mild cognitive impairment and dementia. *Current Gerontology and Geriatrics Research*.

[2] Siegler R. S. (2006). Inter- and intra-individual differences in problem solving across the lifespan. *Lifespan Cognition: Mechanisms of Change*.

[3] Mendonca M. D., Alves L., Bugalho P. (2015). From subjective cognitive complaints to dementia: who is at risk?: a systematic review. *American Journal of Alzheimer's Disease and Other Dementias*.

[4] Ginó S., Mendes T., Maroco J. (2010). Memory complaints are frequent but qualitatively different in young and elderly healthy people. *Gerontology*.

[5] João A. A., Maroco J., Ginó S., Mendes T., de Mendonça A., Martins I. P. (2015). Education modifies the type of subjective memory complaints in older people. *International Journal of Geriatric Psychiatry*.

[6] Jessen F., Wiese B., Bachmann C. (2010). Prediction of dementia by subjective memory impairment: effects of severity and temporal association with cognitive impairment. *Archives of General Psychiatry*.

[7] Rönnlund M., Sundström A., Adolfsson R., Nilsson L.-G. (2015). Subjective memory impairment in older adults predicts future dementia independent of baseline memory performance: evidence from the Betula prospective cohort study. *Alzheimer's & Dementia*.

[8] Pires C., Silva D., Maroco J. (2012). Memory complaints associated with seeking clinical care. *International Journal of Alzheimer's Disease*.

[9] Silva D., Guerreiro M., Faria C., Maroco J., Schmand B. A., De Mendonça A. (2014). Significance of subjective memory complaints in the clinical setting. *Journal of Geriatric Psychiatry and Neurology*.

[10] Mitchell A. J., Beaumont H., Ferguson D., Yadegarfar M., Stubbs B. (2014). Risk of dementia and mild cognitive impairment in older people with subjective memory complaints: Meta-analysis. *Acta Psychiatrica Scandinavica*.

[11] Jonker C., Geerlings M. I., Schmand B. (2000). Are memory complaints predictive for dementia? A review of clinical and population-based studies. *International Journal of Geriatric Psychiatry*.

[12] Baumgart M., Snyder H. M., Carrillo M. C., Fazio S., Kim H., Johns H. (2015). Summary of the evidence on modifiable risk factors for cognitive decline and dementia: a population-based perspective. *Alzheimer's & Dementia*.

[13] WHO Depression is a common illness and people suffering from depression need support and treatment. http://www.who.int/mediacentre/news/notes/2012/mental_health_day_20121009/en/.

[14] Balash Y., Mordechovich M., Shabtai H., Giladi N., Gurevich T., Korczyn A. D. (2013). Subjective memory complaints in elders: depression, anxiety, or cognitive decline?. *Acta Neurologica Scandinavica*.

[15] Del Brutto O. H., Mera R. M., Del Brutto V. J. (2015). Influence of depression, anxiety and stress on cognitive performance in community-dwelling older adults living in rural Ecuador: results of the Atahualpa Project. *Geriatrics & Gerontology International*.

[16] e Silva L. D. S. V., da Silva T. B. L., Falcão D. V. D. S. (2014). Relations between memory complaints, depressive symptoms and cognitive performance among community dwelling elderly. *Revista de Psiquiatria Clinica*.

[17] Holmes-Truscott E., Pouwer F., Speight J. (2014). Further investigation of the psychometric properties of the insulin treatment appraisal scale among insulin-using and non-insulin-using adults with type 2 diabetes: results from diabetes MILES—Australia. *Health and Quality of Life Outcomes*.

[18] Singh-Manoux A., Dugravot A., Ankri J. (2014). Subjective cognitive complaints and mortality: does the type of complaint matter?. *Journal of Psychiatric Research*.

[19] Morgado J., Rocha C., Maruta C., Guerreiro M., Martins I., Morgado J. (2009). Novos valores normativos do mini-mental state examination. *Sinapse*.

[20] Freitas S., Simões M. R., Alves L., Santana I. (2011). Montreal Cognitive Assessment (MoCA): normative study for the Portuguese population. *Journal of Clinical and Experimental Neuropsychology*.

[21] Ginó S., Mendes T., Ribeiro F., Mendonça A., Guerreiro M., Garcia C., Mendonça A., Guerreiro M. (2007). Escala de Queixas de Memória. *Escalas e Testes na Demência*.

[22] Barreto J., Leuschner A., Santos F., Sobral M., Mendonça A., Guerreiro M. (2007). Escala de depressão geriátrica. *Escalas e Testes na Demência*.

[23] Mendes T., Ginó S., Ribeiro F. (2008). Memory complaints in healthy young and elderly adults: reliability of memory reporting. *Aging & Mental Health*.

[24] Holmen J., Langballe E. M., Midthjell K. (2013). Gender differences in subjective memory impairment in a general population: the HUNT study, Norway. *BMC Psychology*.

[25] Instituto Nacional de Estatística (INE) (2011). *Portuguese Official Statistics*.

[26] Mota-Pinto A., Rodrigues V., Botelho A. (2011). A socio-demographic study of aging in the Portuguese population: the EPEPP study. *Archives of Gerontology and Geriatrics*.

[27] Sousa M., Pereira A., Costa R., Rami L. (2015). Initial phase of adaptation of Memory alteration Test (M@T) in a Portuguese sample. *Archives of Gerontology and Geriatrics*.

